# At the Root of Nodule Organogenesis: Conserved Regulatory Pathways Recruited by Rhizobia

**DOI:** 10.3390/plants10122654

**Published:** 2021-12-02

**Authors:** Maria Lebedeva, Mahboobeh Azarakhsh, Darina Sadikova, Lyudmila Lutova

**Affiliations:** 1 Department of Genetics and Biotechnology, Saint Petersburg State University, Universitetskaya emb.7/9, 199034 Saint Petersburg, Russia; darinasadikova@yandex.ru (D.S.); l.lutova@spbu.ru (L.L.); 2 Center for Genetic Technologies, N. I. Vavilov All-Russian Institute of Plant Genetic Resources (VIR), 190000 Saint Petersburg, Russia; 3 Cell and Molecular Biology Department, Kosar University of Bojnord, 9415615458 Bojnord, Iran; mahboobeazarakhsh@kub.ac.ir

**Keywords:** symbiotic nodule development, transcription factors, NIN, LBD16, KNOX, miR172, systemic control of nodulation, CLE, CEP, evolution of legume–rhizobia symbiosis

## Abstract

The interaction between legume plants and soil bacteria rhizobia results in the formation of new organs on the plant roots, symbiotic nodules, where rhizobia fix atmospheric nitrogen. Symbiotic nodules represent a perfect model to trace how the pre-existing regulatory pathways have been recruited and modified to control the development of evolutionary “new” organs. In particular, genes involved in the early stages of lateral root development have been co-opted to regulate nodule development. Other regulatory pathways, including the players of the KNOX-cytokinin module, the homologues of the miR172-AP2 module, and the players of the systemic response to nutrient availability, have also been recruited to a unique regulatory program effectively governing symbiotic nodule development. The role of the NIN transcription factor in the recruitment of such regulatory modules to nodulation is discussed in more details.

## 1. Introduction

Plants have adapted to grow in the nutrient poor soil due to beneficial interactions with soil microorganisms, helping plants enhance nutrient acquisition. In particular, the symbiosis of legume plants with soil nitrogen-fixing bacteria rhizobia supplies plants with biologically available nitrogen. Rhizobia colonize the plant root and induce the formation of symbiotic organs on the root, the nodules, where they reside and differentiate into bacteroids to fix atmospheric nitrogen. The legume–rhizobia symbiosis evolved from a more ancient type of plant endosymbiosis with arbuscular mycorrhiza fungi, which form symbiotic associations with most land plants and help them to uptake more nutrients, in particular more phosphorus, from the soil [1]. In addition to legumes, the non-legume genus *Parasponia* (Cannabaceae) also forms symbiotic nodules with rhizobia [2]. A *Parasponia*–rhizobia symbiosis has been suggested to be evolutionary young and less specialized in comparison to the legume–rhizobia symbiosis. *Parasponia* nodules develop as modified lateral roots with a central vascular bundle and infected cells in the peripheral zone [2]. In addition, a set of species from the Fagales, Cucurbitales, and Rosales orders (collectively known as actinorhizal plants) are able to form the nitrogen-fixing symbiosis with the actinobacterial genus *Frankia*, and their symbiotic organs represent modified lateral roots (LRs) [3]. Plant species capable of forming the nitrogen-fixing symbiosis form so-called nitrogen-fixing clade [4].

In contrast to symbiotic nodules formed by *Parasponia* and actinorhizal plants, developing as modified lateral roots with centrally located vascular bundle, symbiotic nodules of legume plants have a more specialized structure. They represent spherical or cylindrical lateral organs with two or more peripheral vascular bundles converging towards the apical part of the nodule. Nevertheless, a set of data suggests that both symbiotic nodules and lateral roots share common regulators, and the players of the root developmental program have been recruited to control nodule organogenesis (for review see [5]). In addition, other regulatory pathways participate in symbiotic nodule development making the nodule a unique lateral organ. The *NIN* (*NODULE INCEPTION*) gene is specifically induced by rhizobia in inoculated plant roots. It encodes a key transcription factor activating diverse regulatory modules during nodulation [6,7]. In this review, we discuss recently published data on the regulatory modules which have been recruited to nodule organogenesis from other conserved developmental pathways. By “regulatory module”, we mean two or more interacting regulators, i.e., an activator/repressor and its target that could be considered as a single homologous “block” present in different regulatory pathways. Here, we provide a view on the program of symbiotic nodule development as a network of conserved regulatory modules, whose players have well-defined roles in other developmental pathways beyond nodulation. Our review focuses on some striking examples of such regulatory modules which include the players of the root developmental program, the homologues of shoot apical meristem regulators and regulators of flowering transition, as well as systemic regulators of nutrient responses. The recruitment of these pathways to nodulation during the evolution of legumes might have ensured the efficient and fine-tuned control of symbiotic nodule development by a host plant.

## 2. How Rhizobia Govern the Developmental Programs in Legumes: NIN as a Key Regulatory Hub in Nodulation

**Figure 1:**
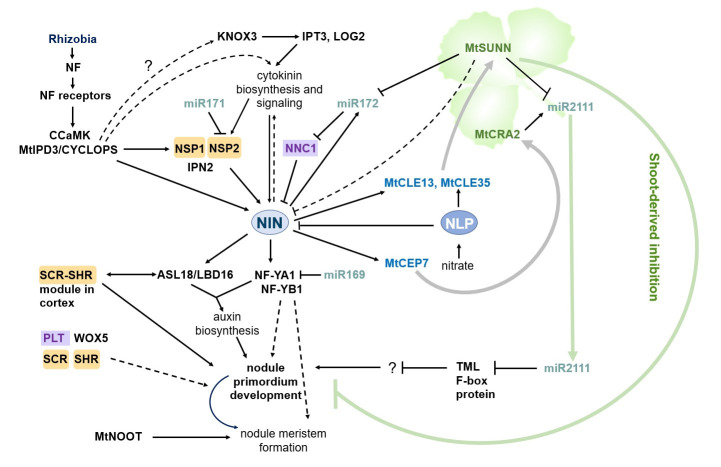
Regulation of symbiotic nodule development in legumes. Rhizobia produce Nod-factors, which activate a signaling cascade leading to phosphorylation of the MtIPD3/LjCYCLOPS TF by CCaMK. MtIPD3/LjCYCLOPS and cytokinin-dependent pathway activate the expression of the *NIN* gene and other genes involved in the legume–rhizobia symbiosis. NSP1 and NSP2 TFs act downstream of MtIPD3/LjCYCLOPS, and NSP1 is able to bind to the promoter of the *NIN* gene. A MYB coiled-coil type transcription factor IPN2 (Interacting Protein of NSP2) interacts with NSP1 and NSP2 to activate *NIN* expression [23,24]. The *NSP2* transcripts are post-transcriptionally regulated by miR171. The KNOX3 TF activates cytokinin biosynthesis genes, *IPT3* and *LOG2*, during nodulation, and other factors contribute to activation of cytokinin biosynthesis at the very early stages of nodulation. The NIN TF directly induces the expression of *NF-YA1* and *NF-YB1* which regulate nodule development and emergence, as well as nodule meristem formation. *NF-YA1* is targeted by miR169. NIN also activates the *ASL18/LBD16* gene involved in the LR developmental program. ASL18/LBD16 forms complex with the NF-Y transcription factors to regulate both LR and nodule development by activating auxin biosynthesis. Other components of the root developmental program, including the PLT, SCR, SHR, and WOX5 TFs are also induced during nodule primordia development. MtNOOT1 and MtNOOT2 establish and maintain indeterminate nodule identity. NIN upregulates the level of miR172 which negatively regulates *NNC1*. NNC1 is а transcriptional repressor interacting with NIN and inhibit NIN activity. Both miR172 and *NIN* expression levels are negatively regulated by the autoregulation of nodulation (AON). NIN directly activates the expression of the *CLE* genes, including *MtCLE13* in *M. truncatula*. In addition to NIN, the NLP TF activates the expression of the nitrate-induced *CLE* genes (*MtCLE35* in *M. truncatula*) in response to the nitrate. The CLE peptides are produced in the root in response to rhizobia inoculation and the nitrate treatment and move to the shoot where they are recognized by their receptors, including the CLV1-like receptor kinase (MtSUNN in *M. truncatula*). Activation of the CLV1-like receptor kinase in the shoot triggers a negative feedback response, which inhibits subsequent nodulation on the root. *MtCEP7* expression is also upregulated by NIN, and it positively regulates nodule development. The CEP peptides involved in nodulation are suggested to move from the root to the shoot, where they are recognized by their receptor (MtCRA2 in *M. truncatula*). MtCRA2-dependent signaling cascade upregulates and MtSUNN-dependent signaling cascade downregulates miR2111, which is a mobile miRNA transported from the shoot to the root. The *TML* transcripts, encoding an F-box-containing protein which negatively regulates nodulation, are the targets of miR2111 in the root. The GRAS TFs are highlighted by orange rectangles, the AP2/ERF TFs are highlighted by violet rectangles.

The formation of nodules is generally initiated by a signaling pathway triggered by Nod-factors—lipochito-oligosaccharide signal molecules secreted by rhizobia (see Figure 1). Perception of Nod-factors by plant receptors induces a signaling cascade, including a calcium-/calmodulin-dependent kinase (CCaMK) [8], which phosphorylates a coiled-coil transcription factor MtIPD3 (INTERACTING PROTEIN OF DMI3)/LjCYCLOPS [9,10,11,12]. In its turn, MtIPD3/LjCYCLOPS activates the expression of the *NODULE INCEPTION* (*NIN*) gene [13] (see Figure 1) which encodes a key transcription factor controlling the subsequent steps of nodulation by regulating both epidermal infection and nodule organogenesis (for review see [7]). A set of components of the Nod-factor signaling cascade, including CCaMK and MtIPD3/LjCYCLOPS, is involved in arbuscular mycorrhizal symbiosis establishment as well [10,11,12]. In this regard, they are considered to be part of a common symbiotic (SYM) signaling pathway [1]. Transcription factors (TFs) of the GRAS family, NSP1 and NSP2 (NODULATION SIGNALING PATHWAY) operate downstream of CCaMK and activate the expression of other genes necessary for the legume–rhizobia symbiosis [14,15] (Figure 1). A NSP1–NSP2 complex, where NSP1 is a DNA binding protein, whereas NSP2 lacks a DNA binding domain, binds to a specific promoter region of rhizobia-induced genes, present in the *NIN* gene, in particular [14]. In addition to their role in the legume–rhizobia symbiosis, NSP1 and NSP2 are also involved in the arbuscular mycorrhizal symbiosis [16,17], where they regulate strigolactone biosynthesis required for mycorrhization [18].

Activation of cytokinin biosynthesis genes and subsequent cytokinin accumulation are observed early in response to rhizobial inoculation/Nod-factor treatment [19,20,21]. Cytokinin is a key positive regulator of cortical cell division leading to nodule primordium development, and at the same time, it negatively regulates rhizobial infection (for review see [22]). Despite the fact that much knowledge has been accumulated on cytokinin biosynthesis and response gene action during nodulation in legume plants (see [22]), we still do not know definitively which factor activates cytokinin biosynthesis and response genes at the very early stages of Nod-factor-induced signaling cascade.

Downstream of Nod-factor-induced signaling cascade and cytokinin, the expression of the *NIN* gene is activated [14] (see Figure 1). NIN is a key TF in nodulation, and it acts as a regulatory hub coordinating different regulatory pathways in nodulation [6,7]. In general, two spatially distinct programs are activated after the initial step of Nod-factor recognition by plant receptors: an epidermal program associated with the rhizobial infection and nodule primordium formation from pericycle and root cortical cells (for review see [25]). NIN regulates both of these programs. The expression of *NIN* is observed in the epidermis, where infection takes place, and also in the inner cell layers, including the pericycle and cortex cells, where nodule primordium is initiated [26]. Spatiotemporal pattern of *NIN* expression is determined by the presence of different *cis*-regulatory elements in its promoter [13,26]. *NIN* expression in the epidermal cells is essential for infection thread formation [27]. In *M. truncatula*, the CYCLOPS-binding site in the *NIN* promoter located about 3 kb upstream of the start codon drives *NIN* expression in epidermis, and its deletion diminishes infection thread formation [26]. A distal promoter element including putative cytokinin response regulators (RRB) binding sites (named the CE region) is responsible for the induction of *NIN* expression in the inner cell layers necessary for nodule primordium formation is required [26]. The presence of the CE region in the *NIN* promoter was found to be specific for *NIN*-related genes in legumes, whereas the CYCLOPS-binding sites occur in the *NIN* promoters of legumes and non-legume nodulating plants, including *Parasponia* and actinorhizal plant *Casuarina* [7]. Cytokinin-dependent regulation of *NIN* expression is suggested to be legume-specific. Therefore, the occurrence of the CE region in the distal promoter of the *NIN* gene might be considered as an evolutionary important event leading to the legume–rhizobia symbiosis establishment [7,26].

Initially, NIN was identified as a nodulation-specific TF [27,28], and later the homologues of NIN belonging to a large family of NIN-like proteins (NLP) were described in both legume and non-legume plants as important regulators of nitrate-inducible gene expression [7,29]. NLP TFs possess the RWP-PK DNA binding domain in their C-terminal region which binds to NRE (nitrate-responsive cis-element) in the target genes [29]. The N-terminal region of the NLP proteins is responsible for the activation of gene expression in response to the nitrate [30]. In *A. thaliana*, AtNLP7 is accumulated in the nucleus in the presence of the nitrate, where it binds to NRE and activates the expression of nitrate-responsive target genes. Such activation and nuclear accumulation of AtNLP7 in response to the nitrate is mediated by phosphorylation of Ser-205 in AtNLP7 by Ca^2+^-sensor protein kinases (CPKs), which are induced by calcium signaling triggered by the nitrate [30,31]. In contrast to other NLP proteins, NIN does not respond to the nitrate signal, and such a loss of nitrate-responsiveness was suggested to be an important evolutionary event necessary for the establishment of the legume–rhizobia symbiosis [32]. According to phylogenetic and microsynteny analysis, *NIN* orthologues are present in all the nodulating species as well as in the genomes of species outside the nitrogen-fixing clade [33]. However, in the genomes of non-nodulating species from the nitrogen-fixing clade, multiple independent evolutionary events having led to the loss of *NIN* function in non-nodulating species were found [33]. This highlights the significance of NIN as a master regulator necessary for the successful nitrogen-fixing symbiosis establishment.

It was found that the NIN and NLP proteins are capable of binding to the same *cis*-regulatory elements in the target genes [34]. Moreover, NIN and NLP interact through their N-terminal PB1 domains to form heterodimers on *cis*-regulated DNA regions [34,35]. Despite of fact that that binding sites for the NIN and NLP TFs generally overlap and share a consensus sequence pattern with semi-palindromic structures, in *L. japonicus,* NIN and NLP have different DNA-binding specificities, where the NIN-specific binding sites were found to be less palindromic [34]. In *L. japonicus*, LjNLP4–LjNIN interaction reduces the chances of DNA binding by the LjNIN homodimers, and NLP could reduce the expression of the NIN-activated target genes. Therefore, in the presence of the nitrate, NLPs are accumulated in the nucleus and reduce NIN-mediated activation of symbiosis-related target genes, which could account for the nitrate-mediated inhibition of the symbiosis [34]. On the other hand, NIN alone has a weaker ability to induce the expression of nitrate-responsive genes activated by the NLP homodimers, whereas the formation of the NIN–NLP heterodimers reduced the induction level of nitrate-dependent gene expression brought by NLP [34]. However, among the NIN and NLP target genes, the *CLE* (*CLV3/EMBRYO-SURROUNDING REGION*) genes involved in autoregulation of nodulation contain both the NLP-specific and NIN-specific binding sites, and, therefore, they are upregulated by both the nitrate and rhizobia-induced signaling cascade (see Section 6). Therefore, the NIN-binding sites might have evolved as modified NRE, and the occurrence of new NIN-specific binding sites in the target gene promoters resulted in their recruitment to the nodulation program and accompanied the establishment of the legume–rhizobia symbiosis.

The NIN TF regulates the expression of a set of genes involved in nodule development (see Table 1, Figure 1). Among the direct targets of NIN, there are: *RPG* (*RHIZOBIUM-DIRECTED POLAR GROWTH*), *NPL* (*NODULATION PECTATE LYASE1*) and other genes involved in infection thread formation [6,36], the genes encoding the NF-YA1 and NF-YB1 (Nuclear Factor-Y A1 and B1) TFs which are essential for both infection and nodule primordia development [37,38], the *CRE1* (*CYTOKININ RESPONSE 1*) gene encoding cytokinin receptor in *M. truncatula* [39], the *LBD16* (*LOB-DOMAIN PROTEIN 16*) gene [40,41], as well as microRNA miR172c [42]. Moreover, NIN directly activates the expression of the *CLE* genes, encoding regulatory peptides inhibiting nodulation by a shoot-dependent negative feedback mechanism [43]. At the same time, NIN activates the expression of the *MtCEP7* gene encoding a regulatory peptide of the CEP (C-TERMINALLY ENCODED PEPTIDEs) family that positively regulates nodule formation [44].

## 3. Players of the Root Developmental Networks in Nodule Organogenesis

Nodule primordium formation is initiated in the inner cell layers of the root. The cell layers where the initial cell divisions take place differ between the two types of symbiotic nodules: determinate and indeterminate ones (see [66]). In plants with indeterminate nodules (such as *Medicago truncatula*, pea and clover), initial cell divisions are observed in pericycle cells followed by the reactivation of the endodermal and inner cortex cells [67,68]. Specifically, in *M. truncatula*, the meristem of nodule primordia is derived from the third cortical cell layers, whereas the inner cortical cells, the endodermal, and pericycle cells contribute to the basal part of symbiotic nodule [68]. In plants with the determinate type of nodules (*Lotus japonicus*, soybean and common bean), cell divisions occur in the middle or outer cortex cells, leading to the formation of determinate nodules with a spherical shape that lack a persistent nodule meristem (see [66]).

Reactivation of cell divisions in the root also takes place during lateral root (LR) primordium development. In model species *A. thaliana* and other members of *Brassicaceae* family, only pericycle cells are mitotically activated during LR primordium formation. In the majority of other plant species, analyzed by Xiao et al., cortical and endodermal cell divisions were observed during LR formation [69]. Moreover, in *M. truncatula*, pea, and *Cucurbitaceae* species the cells derived from endodermis and cortex contribute to the LR primordium [69,70,71].

Auxin is a key hormone which triggers LR primordium formation. The LR founder cells, a group of the xylem pericycle cells where first divisions leading to LR primordium development occur, are characterized by an enhanced auxin maximum. Local auxin accumulation in the pericycle cells of *Arabidopsis* is required for LR formation [72]. Auxin activates the expression of key regulators of LR primordium initiation, including genes for the GATA23 and LBD16 TFs [73,74]. Genes from the auxin-regulated transcriptional network encoding WUSCHEL-RELATED HOMEOBOX 5 (WOX5) [75,76], the PLETHORA (PLT) TFs [77,78], and SHORTROOT (SHR) [79,80] known as regulators of the root apical meristem (RAM) are also induced during LR formation.

Auxin also was found to play a crucial role in nodulation by controlling both rhizobial infection and cell divisions during nodule primordia formation (for review, see [81]). Exogenous auxin treatment (specifically, 2,4-D) is able to induce nodule-like structures (NLS) in the root in both legume and non-legume plants [82,83,84,85]. Auxin-induced NLS in rice demonstrated the enhanced induction of the *PLT*, *WOX5,* and *WOX11* genes involved in the lateral root developmental program, whereas rice orthologs of the nodulation-related *NSP2* gene and other players of nodule symbiotic pathway were not activated in auxin-induced NLS in rice [86]. Therefore, auxin-induced NLS are common for both nodulating and non-nodulating species, and the ability of exogeneous auxin to induce nodule-like structure does not underlie the specific cellular response of legume plants to rhizobia.

### 3.1. Genes Controlling the Initiation of Lateral Root Development and Their Role in Nodule Organogenesis

In *A. thaliana*, a set of transcriptional regulators is required for the early steps of lateral root (LR) primordium initiation. Among them, there are the GATA23 and LATERAL ORGAN BOUNDARIES-DOMAIN 16 (LBD16)/ASYMMETRIC LEAVES2-LIKE 18 (ASL18) TFs, which are activated by the auxin signaling module in the LR founder cells [73,74]. The LBD/ASL proteins belong to the AS2 (ASYMMETRIC LEAVES2)/LOB (LATERAL ORGAN BOUNDARIES-DOMAIN) family, and they are involved in various aspects of plant development playing a crucial role in defining organ boundaries [87]. LBD16/ASL18 is activated specifically in the LR founder cells by AUXIN RESPONSE FACTOR (ARF) 7 and ARF19, and its activation is required for the establishment of the asymmetry in the LR founder cells before cell division [88].

Recently, the *LBD16/ASL18* gene was found to be important for the early steps of nodule primordium formation in both *M. truncatula* and *L. japonicus* [40,41]. The expression of the *LBD16/ASL18* gene was observed at the sites of both LR and nodule initiation, and its expression was induced by auxin [41]. In *L. japonicus*, knock-out of the *LBD16/ASL18* gene resulted in lower LR densities and reduced number of nodules with reduced size [40]. In *M. truncatula*, roots overexpressing *LBD16* demonstrated extensive root curling and initiation of ectopic root primordia [41]. Co-overexpression of the *LjLBD16/LjASL18* and NF-Y subunit genes, which are also known as the direct targets of NIN, increased lateral root densities and also resulted in ectopic cell divisions in the root leading to bump formation [40]. LjLBD16/LjASL18 is able to interact with NF-Y transcription factors both in vivo and *in planta* [40], suggesting that LBD16/ASL18 could act in a complex with the NF-Y transcription factors to promote both lateral root and nodule development.

Initially, the *MtNF-YA1* gene (previously known as *MtHAP2-1*) was described in *M. truncatula* as an essential regulator of nodule meristem development and rhizobial infection [38,46]. In *L. japonicus*, the roles of the *NF-YA1* and *NF-YB1* genes in nodule development have also been reported, and these genes regulate LR development as well [37]. LjNF-YA1 stimulates the expression of the *SHORT INTERNODES/STYLISH* (*STY*) genes encoding transcription factors which are required for nodule emergence and differentiation and which activate the expression of auxin biosynthesis genes [89,90]. Moreover, the miR169-regulated *AtNF-YA2* and *AtNF-YA10* genes in *A. thaliana* are involved in root growth and LR development, suggesting that the miR169-NF-YA module is a basic regulator of the root developmental program [47]. Interestingly, miR169 also might be involved in long-distant signaling in plant for reporting P or N status: miR169 was found in rapeseed (*Brassica napus*) phloem sap and its abundance decreased during N and P limitation and significantly increased during N-replete growth [91]. Therefore, it was suggested that miR169 could also be involved in the systemic regulation of nodulation depending on nitrogen availability (see also Section 6).

In *L. japonicus* and *M. truncatula*, the expression of *LBD16/ASL18* in response to rhizobia inoculation is activated by the NIN TF, indicating that the NIN transcription factor is responsible for the recruitment of the *LBD16/ASL18* gene to the nodule organogenesis program. Soyano et al. found the NIN-binding sites (NBS) located in the *ASL18* introns (one NBS-S1 in the *LjASL18b* gene or both NBS-S1 and NBS-S2 in the *LjASL18a* gene) [40]. Importantly, NBS-S1 and its flanking sequences were found to be conserved in legume plants, but were not observed in the *LBD16/ASL18* genes in non-legume plants [40]. Therefore, the occurrence of NBS in the *LBD16/ASL18* genes in legume plants might play an important role during the evolution of the legume–rhizobia symbiosis, and recruitment of LBD16/ASL18, a LR developmental regulator, to nodule signaling pathway by the NIN TF is responsible for the activation of cell divisions in root cortex, which makes possible nodule primordia formation [40,41].

### 3.2. Genes Controlling Root Apical Meristem Development and Maintenance and Their Role in Nodule Organogenesis

In the developing and mature symbiotic nodules, the activity of regulators involved in root apical meristem (RAM) development and maintenance has also been reported [92,93,94,95]. In the RAM, the stem cell niche is maintained due to the activity of a set of TFs, which act in tight connection with auxin, including the WUSCHEL-RELATED HOMEOBOX 5 (WOX5), PLETHORA (PLT), SHORTROOT (SHR), and SCARECROW (SCR) proteins. The *WOX5* gene, which encodes a homeodomain-containing TF, is expressed in the quiescent center of the RAM, and its activity is required for root stem cell maintenance and inhibition of stem cell differentiation [75]. In *A. thaliana*, *WOX5* regulates auxin biosynthesis in the quiescent center [96], and auxin maximum in the RAM is known to determine the position of the stem cell niche [97]. Auxin also regulates the expression of four *PLT* genes encoding TFs that belong to the AINTEGUMENTA-LIKE (AIL) subgroup of the APETALA2/Ethylene Responsive Factor (AP2/ERF) family [77]. The PLT TFs are required for RAM maintenance and root stem cell niche formation. The PLT proteins form a gradient in the root apex, which is essential for proper cell proliferation and differentiation in the apical part of the root, and their highest concentration is observed in the root stem cell niche [98]. PLTs were shown to control the expression of the *PIN* genes, required for the proper auxin distribution in the root, as well as other regulators of RAM, including the *SHR* gene encoding a TF from the GRAS family [99]. SHR regulates the expression of the *SCR* gene, which also encodes a GRAS TF, and they both are required for RAM development and maintenance as well as for the proper radial patterning of the root [100].

In *M. truncatula*, homologues of the WOX5, PLT, SHR, and SCR TFs were shown to participate in the nodule developmental program [92,93,94,95]. The expression of the *WOX5* gene was observed in the nodule primordia and, at the later stages, in the tips of vascular bundles of mature nodules [92]. Moreover, it was shown that the tips of the nodule vascular bundles also exhibit the maximum of auxin response visualized by DR5 reporter activity [101]. In addition, other homologues of RAM regulators, including *MtPLT1* and *MtPLT2*, are also expressed in these sites [94]. The regions corresponding to the tips of vascular bundles in the nodule apex are referred to as the nodule vascular meristem (NVM) [94]. Based on the expression patterns of the specific markers of the “quiescence center”, it could be speculated that the NVM represents a domain similar to the organizing center of RAM. Interestingly, the RAM-specific nature of the NVM is also manifested by the phenotype of *M. truncatula* and pea mutants defective in the *MtNOOT1*,*2/PsCOCH* genes, which develop ectopic root from the NVM [102,103,104]. In *M. truncatula*, the *Mtnoot1 noot2* double mutant demonstrated the complete loss of nodule identity with nodule-to-root homeosis [104]. Therefore, the *MtNOOT1*,*2* and *PsCOCH* genes control the activity of the NVM and define nodule identity. In addition to its role in the NVM, the *MtNOOT1* gene also controls the size of the RAM by defining the position of the boundary region between the apical meristem and differentiation zone [105]. The primary roots of the *Mtnoot1* mutant demonstrated delayed xylem cell differentiation suggesting that MtNOOT1 promotes root vasculature differentiation [104].

The close homologues of the *MtNOOT1*,*2/PsCOCH* genes in *A. thaliana* are the *BLADE-ON-PETIOLE 1* (*BOP1*) and *BOP2* genes encoding co-transcriptional factors suppressing meristematic activity in the developing lateral organs as well as other developmental processes related to plant organ boundary regulation [106]. It was reported that BOP1 and BOP2 positively regulate the expression of the *LBD/ASL* genes and downregulate the expression of the *KNOX* genes in the developing leaf primordia, which encodes homeodomain transcription factors necessary for shoot meristem maintenance [106]. Interestingly, in *M. truncatula*, among three *MtKNOX* genes upregulated during nodulation, the expression levels of *MtKNOX9* and *MtKNOX3* were downregulated in the *Mtnoot1/Mtnoot1 noot2* nodules, whereas *MtKNOX5* expression was induced [104]. Therefore, like BOPs in *A. thaliana*, their homologues in *M. truncatula* also regulate the expression patterns of the *KNOX* genes. Since the homologues of the *LBD/ASL* genes are also involved in symbiotic nodule development, it would be interesting to study whether NOOT-BOP-COCH-LIKE (NBCL) could also regulate their expression in the developing nodule.

The involvement of root apical meristem-related genes in nodulation was shown for *M. truncatula* and pea, which form nodules of the indeterminate type with persistent meristem. The roles of these genes in the development of determinate nodules have not been studied yet. Interestingly, the mutation in *LjNBCL1*, an ortholog of the *NBCL* gene in *L. japonicus*, also results in ectopic roots arising from the NVM, suggesting that the NBCL genes function in both indeterminate and determinate nodules through the maintenance of nodule vascular bundle identity [107]. A similar phenotype was also observed when *Phaseolus vulgaris* plants, forming determinate nodules, were inoculated by rhizobia strains having mutations in the bacterial genes controlling lysine, purine, and pyrimidine biosynthesis [108]. Therefore, the suppression of the lateral root identity program in the cells of the NVM can be overpassed in both indeterminate and determinate types of nodules, which leads to the formation of ectopic roots from the developing nodule.

### 3.3. Endodermal and Cortical Cell Fate Regulators in Nodule Organogenesis

In addition to their role in root apical meristem, the SCR and SHR TFs are key regulators of the root radial patterning that are responsible for the cortical and endodermal cell differentiation in the root [100]. In *Arabidopsis*, the *SHR* gene is expressed in the stele cells of the root, and its protein product moves to the adjacent cell layers, including endodermis, quiescent center, the cortical endodermal initials, and cortical endodermal daughter cells, where the SHR TF activates the expression of the *SCR* gene. SHR together with SCR activates the expression of a D-type cyclin gene, CYCD6;1, which was shown to promote the asymmetric formative cell divisions separating the ground tissue into two different cell layers, the endodermal and cortical ones [109]. Ectopic *SHR* expression under the *SCR* promoter increased the number of endodermal cell layers in *Arabidopsis* [110]. Recently, it was shown that the SHR–SCR regulatory module is essential for legume-specific cortical cell divisions during nodulation [95].

In contrast to *Arabisopsis*, in *M. truncatula* and other legumes, the *SCR* gene is expressed not only in endodermis, but also in the root cortical cells and to a lesser degree in the root epidermal cells. Moreover, the accumulation of the SHR protein was also observed in the cortical cells in legumes [95]. The presence of *SCR* expression in the root cortex was associated with the occurrence of two *cis*-regulatory elements located close to each other only in the *SCR* promoters from legumes, but not in the ones from the species outside the nitrogen fixing clade. Furthermore, in *M. truncatula* the homologues of both the *SHR* and *SCR* genes are required for cortical cell divisions leading to nodule development, where the MtSHR1/2 protein levels are increased in the epidermal and cortical cells in response to rhizobial inoculation, and MtSHR1/2 subsequently activates the expression of the *MtSCR* gene [95]. *MtSHR1* overexpression induces cortical cell divisions leading to the formation of nodule-like structures without rhizobial inoculation, which requires the functional *MtSCR* gene, but does not depend on the Nod-factor signaling components including NSP1, NSP2, and NIN. The SCR–SHR activity in the root cortex of legume plants was shown to be prerequisite for rhizobia-induced cortical cell divisions, as well as for the formation of nodule-like structures induced by cytokinin treatment or the ectopic expression of the *NIN* gene, suggesting that the SCR–SHR regulatory module acts downstream of cytokinin and NIN in nodulation (see Figure 1). These data suggest the involvement of the root-specific SCR–SHR regulatory module in the early steps of nodule primordium development which was recruited for the nodulation due to the broader patterns of *SCR* and *SHR* expression in legume roots encompassing the cortical and epidermal cells [95].

## 4. Cytokinin and the Homologues of Shoot Apical Meristem Regulators in Nodule Organogenesis

Cytokinin is as a key hormone stimulating shoot apical meristem development and maintenance (see [111]). In the shoot apical meristem, cytokinin amount is increased due to the local activation of the cytokinin biosynthesis genes by class I KNOX transcription factors [112,113]. Cytokinin is also a key positive regulator of nodule primordium development. Exogenously applied cytokinin stimulates the formation of nodule-like structures on the roots of legume plants [114,115]. In addition, a gain-of-function mutation in cytokinin receptor genes resulted in spontaneous nodule formation [116,117]. Cytokinin-response regulators activate the expression the *NSP2* and *NIN* genes, which are also induced by the rhizobia-induced signaling cascade, suggesting the convergence of the cytokinin and symbiosis-induced pathway [26,118]. A key role of cytokinin in nodule organogenesis has been supported thoroughly by a set of genetic studies (for review see [22]).

At the same time, cytokinin is considered to be a negative regulator of LR development. Mutants with reduced cytokinin production and response possessed larger number of LRs [50,51], whereas exogenous cytokinin application represses LR initiation, influencing cell division pattern in the root [51,119]. Moreover, the increased cytokinin response is observed between developing LR primordia, suggesting that cytokinin is important for LR spacing along the main root, mediating lateral inhibition of new LR primordia emergence in the adjacent root pericycle cells [120]. However, in contrast to LR development and other developmental processes, such as shoot apical meristem development and maintenance where cytokinin and auxin act antagonistically [121], these two hormones cooperatively regulate nodule primordia development in legume plants.

Like auxin, exogenous cytokinin application was shown to induce nodule-like structures [114,115]. However, unlike auxin, the cytokinin application induces NLS only in nodulating species, including legume plants and *Parasponia* [122]. The ability of exogenously applied cytokinin (specifically, 6-BA) to induce cortical cell divisions was suggested to be an important feature underlying the ability of a plant to form root nodules in response to the signals produced by rhizobia [122]. In several studies, pseudonodules induced by cytokinin demonstrate the expression of the early nodulation genes, like *ENOD40* [123,124] and *NIN* [115]. Moreover, *L. japonicus* mutants defective in *NIN* were not able to form pseudonodules in response to cytokinin treatment, which positions cytokinin action upstream of this symbiotic regulator in the control of NLS formation and highlights its requirement for the cytokinin-mediated activation of root cortical cells [115]. Therefore, cytokinin is an important positive regulator of nodule primordium development, and its action determines the specificity of the nodulation program in legume plants, whereas auxin and auxin-regulated transcriptional regulators are common for both lateral root and nodule primordium development.

Cytokinin biosynthesis gene *LjIPT2* (*ISOPENTENYL TRANSFERASE 2*) and *LjLOG4* (*LONELY GUY 4*), which regulate the production of active cytokinin bases from their nucleotide precursors, as well as *MtIPT2* and *MtIPT4* in *M. truncatula*, were found to be induced early in response to rhizobial inoculation/Nod-factor treatment [19,20,21]. In *L. japonicus*, *LjIPT2* and *LjLOG4* were found to contribute to the first rapid cytokinin accumulation in root cortex leading to nodule primordia development [21]. In addition to this first wave of cytokinin biosynthesis in the root cortex, activation of other members of *IPT* and *LOG* families occurs later during nodule primordium development [125,126,127], which is important for the subsequent stages of nodule organogenesis. Among these genes, there are *MtIPT3* and *MtLOG2* in *M. truncatula*, and for them the direct activation by the KNOX3 (KNOTTED1-LIKE HOMEOBOX 3) TF has been suggested in our previous studies [126,128]. The KNOX proteins are homeodomain containing TFs from the TALE (Three amino acid loop extension) superclass of homeobox proteins, which are subdivided into two classes, class I and class II [129]. The class I KNOX TFs are the key regulators of shoot apical meristem development and maintenance [130]. They maintain SAM (shoot apical meristem) activity by inducing the expression of the *IPT* genes [112,113]. This leads to a local increase of cytokinin in the SAM, where cytokinin is known as the key regulator of cell proliferation [131,132]. The class II KNOX genes are broadly expressed in differentiating tissues and mature organs, being suggested to play the roles antagonistic to the action of the class I KNOX genes [133]. MtKNOX3 is a member of the class II KNOX TFs in *M. truncatula* [134]. Previously, we found that it is involved in the symbiotic nodule development by stimulating the expression of cytokinin biosynthesis genes during nodulation [128]. Down-regulation of the *MtKNOX3* gene by RNAi resulted in a decreased expression of *MtLOG2* and *MtIPT3*, as well as the *MtRR4* gene, a cytokinin response gene in *M. truncatula* [128]. Moreover, we found that the MtKNOX3 homeodomain directly bound to the *MtIPT3* and *MtLOG2* regulatory sequences *in vitro* [126], suggesting the direct activation of cytokinin biosynthesis genes by the MtKNOX3 TF. Together these findings suggest that MtKNOX3, a class II KNOX TF, is involved in the KNOX-cytokinin regulatory module as it was previously shown for the class I KNOX TFs acting in the SAM. Interestingly, the KNOX-IPT regulatory module was also found to be involved in sporophyte axis extension in the moss *Physcomitrella patens*, and it was suggested that such pre-existing KNOX-cytokinin regulatory module was recruited later into vascular plant shoot meristems during evolution [135]. In this connection, the KNOX3 TF with its *IPT3* and *LOG2* target genes involved in cytokinin biosynthesis provides an example of “shoot-related” conserved regulatory module that has been co-opted to regulate cytokinin biosynthesis in symbiotic nodules.

It should be mentioned that in contrast to our findings, Di Giacomo et al. proposed an alternative role of *MtKNOX3* and other class II KNOX TFs in nodulation [136]. In the study by Di Giacomo et al., the simultaneous down-regulation of the *MtKNOX3* gene and three other class II *MtKNOXs* (*MtKNOX5*, *9*, *10*) resulted in the decreased expression of cytokinin response gene *MtRR4* [136], as it was observed for MtKNOX3-RNAi by Azarakhsh et al., 2015 [128]. However, in addition to this, the expression of the *MtEFD* (*ETHYLENE RESPONSE FACTOR REQUIRED FOR NODULE DIFFERENTIATION*) gene was also decreased in the transgenic roots with down-regulation of class II *MtKNOXs*. The MtEFD transcription factor is a negative regulator of nodulation known to be responsible for the activation of the *MtRR4* gene [137]. *MtRR4* is a primary cytokinin-activated gene, and at the same time, it is a member of the Response Regulator A (RRA) gene family, encoding negative regulators of cytokinin signaling [138]. This allowed the authors to suggest that MtKNOX3 together with other class II KNOX TFs inhibited cytokinin signaling through the activation of the EFD/RR4 regulatory module playing a negative role in nodulation [136]. However, the direct activation of the *MtEFD* gene by class II KNOX TFs has not been studied yet. Therefore, these data suggest a more complex role of MtKNOX3 and other class II MtKNOX TFs during nodulation. It should be noted that cytokinin itself have multiple functions in nodulation. In addition to its positive roles in nodule primordium development, cytokinin negatively regulates infection thread formation in the root epidermis [139] and is also involved in later stages of nodulation and nitrogen fixation [140,141]. Moreover, cytokinin was found to mediate shoot-derived inhibition of nodulation in *L. japonicus* [142].

Cytokinin was found to be important for *NIN* activation in legume plants, and the presence of the CE region in the *NIN* promoter was found to be specific for *NIN*-related genes in legumes [7,26]. In addition, it was found that NIN itself activates the cytokinin signaling in the root. NIN directly binds to the promoter of cytokinin receptor gene *CRE1* and activates its expression in the cortex [39]. Therefore, NIN is also responsible for the increasing of cytokinin response in rhizobia-inoculated roots, which may account for the activation of cell divisions leading to nodule primordium development.

## 5. Homologues of Flowering-Related Genes and Their Role in Nodule Organogenesis

The NIN TF is also involved in the evolutionary conserved regulatory module known as NIN- miRNA172- NNC1 [42] (see Figure 1). NIN activates the expression of miR172c [42], a member of the evolutionary conserved microRNA family, which is known to target *APETALA2* (*AP2*) and the homologous genes—well-known regulators of flowering time and flower development [53]. In soybean, miR172c is upregulated in response to rhizobia inoculation and nodule development, and its overexpression increased the number of the infection foci and the nodule primordia [42].

The target of miR172c is the *NNC1* (*NODULE NUMBER CONTROL 1*) mRNA encoding an AP2-like transcriptional repressor. Its closest homologue in *A. thaliana* is TOE1 (TARGET OF EAT1), which represses the transcription of the *FLOWERING LOCUS T* (*FT*) gene (known as florigen) by binding to its promoter and inhibiting the action of its transcriptional activators [143]. NNC1 is a negative regulator of nodulation, and *NNC1* knock-down by RNA-interference increased nodule number in soybean. Moreover, there is evidence that NNC1 directly interacts with NIN, thereby preventing the activation of its target genes [42].

Specifically, NNC1 represses the activity of the early nodulin *ENOD40* gene, which is proposed to account for the NNC1 repressive effect on nodulation. Moreover, NNC1 also acts as a repressor of the *GmRIC1* and *GmRIC2* genes, encoding rhizobia-induced CLE peptides triggering AON in soybean (see Section 6). The NIN-binding sites (NBS) within the promoters of these genes were enriched for the NNC1 protein, and since NIN physically interacts with NNC1, it was proposed that NNC1 prevents activation of the *GmRIC1* and *GmRIC2* genes by competitive binding to NBS and by forming a complex with NIN [42]. In addition, AON activation inhibits the expression of NIN and miR172c by a negative feedback mechanism (see Figure 1). Therefore, the NMN regulatory module coordinates the nodulation and AON regulatory pathways in soybean [42].

Recently, it was shown that in addition to nodulation, the miR172c-NNC1 module also regulates root development in response to the salt stress [52]. *NNC1* knockdown via RNAi resulted in the increased root tolerance to the salt stress, whereas *NNC1* overexpression enhanced salt sensitivity of soybean roots, suggesting that *NNC1* is a negative regulator of root tolerance to the salt stress in soybean. Therefore, the miR172-NNC1 regulatory module has a broad role in plant development and stress response, and it was recruited to nodulation program to orchestrate nodule development by repressing the activity of the NIN TF, a key integrator of different regulatory pathways in nodulation.

In addition to the evolutionary conserved miR172, another conserved microRNA, miR156, acts as its antagonist in the control of flowering transition in plants, as well as in other developmental processes [144]. The targets of miR156 are the transcripts of the genes encoding the SQUAMOSA promoter-binding protein-like (SPL) TFs, which are known as positive regulators of flowering [145]. In contrast to miR172, the amount of which increases with the age of a plant, miR156 level in plants is more abundant at the juvenile stage and decreases with plant aging [146]. Therefore, the level of these two miRNAs is considered to be a “molecular timer” of the age of a plant and determines developmental phase transitions in plants [146]. Interestingly, the role of miR156 in nodulation has also been reported [147,148]. In alfalfa, the effect of miR156 overexpression on nodulation was genotype-dependent: it either increased or had no effect on the number of nodules [148]. On the contrary, the ectopic expression of miR156 in *L. japonicus* reduced nodule numbers, as well as resulted in a decreased expression of a set of nodulation-related genes, including *CYCLOPS*, *NSP1*, and *NIN* [147]. Moreover, in soybean miR156 also reduced nodulation, and its level was negatively correlated with miR172 levels throughout nodule development, suggesting that miR156 might negatively regulate miR172 expression [149]. Therefore, in addition to flowering control, the antagonistic action of the two conserved miRNAs, miR172 and miRNA156, is also manifested in the regulation of symbiotic nodulation.

## 6. Players of Systemic Response to Nutrient Availability and Their Role in Nodulation

The development of symbiotic nodules is regulated by a systemic mechanism known as the autoregulation of nodulation (AON), which controls nodule number in legume plants. The key components of the AON are root-derived CLE (CLV3/EMBRYO-SURROUNDING REGION) peptides moving from the root through the xylem to the leaf where they activate a signaling cascade, which inhibits nodulation [54,55,56]. The CLE peptides represent a group of post-translationally modified regulatory peptides, which are important regulators of plant meristem maintenance, cell differentiation, early embryogenesis, and other developmental processes (for review, see [150]). Some members of the CLE peptide family are able to mediate systemic responses in plants via long-distance transport through the xylem ([54,56,151,152,153], reviewed in [154])

In AON, the CLE peptides are perceived by the CLAVATA1 like Leucine-rich repeat (LRR) receptor kinase (NODULE AUTOREGULATION RECEPTOR KINASE (NARK) in soybean, HYPERNODULATION ABERRANT ROOT FORMATION 1 (HAR1) in *L. japonicus*, SUPER NUMERIC NODULES (SUNN) in *M. truncatula*). Mutations in the corresponding genes cause a supernodulating phenotype [155,156,157]. The CLV1-like kinase genes involved in the AON are the closest relatives of the AtCLV1 kinase in *A. thaliana* which controls the stem cell maintenance in the SAM [158] by perceiving the CLAVATA3 (CLV3) signaling peptide, a member of the CLE peptide family [155,156,157]. In soybean, the GmNARK kinase involved in the AON is a paralogue of the GmCLV1A kinase, which acts in the SAM maintenance [158]. This suggests that the AON-related CLV1-kinases have evolved due to duplication and neodiversification of homologous CLV1-like kinases [159]. Accordingly, the genomic region where the *MtSUNN* gene is located is not syntenic with the region including the *AtCLV1* gene, suggesting that MtSUNN is not a direct ortholog of the AtCLV1 kinase [157].

In addition to its function in the SAM, the CLV1 receptor kinase also functions in the primary root meristem as well as during LR formation, where CLV1 is able to interact with different co-receptors to perceive various CLE peptide ligands [61,160]. The CLE peptides triggering the AON belong to the group which also includes the AtCLE1-AtCLE7 peptides of *A. thaliana* [161,162]. It was found that AtCLE1/3/4/7 from these groups is activated under nitrate deficiency and the overexpression of these genes inhibits lateral root development [61]. Since such an inhibitory effect was abolished in the *Atclv1* mutant, it was suggested that the AtCLE1/3/4/7 peptides inhibit LR development under nitrogen deficiency by acting through the AtCLV1 kinase in the root [61]. Therefore, the CLE peptides triggering the AON through the CLV1-like kinase in the shoot are homologous to the nitrate-regulated CLEs in *A. thaliana*, suggesting that the AON mechanisms might have evolved based on the system which controls root architecture in response to nitrate availability in plants.

Legume *CLE* genes involved in the AON differ in the way their expression is activated. In particular, in *L. japonicus LjCLE-RS 1–3* (*CLE-ROOT SIGNAL*) are activated by rhizobia, whereas *LjCLE-RS 2*,*3* are also activated in response to the nitrate treatment [56,163]. In *M. truncatula*, *MtCLE12* and *MtCLE13* are activated by rhizobia-induced signaling cascade [54] and are specific to nodulation, whereas *MtCLE35* is induced by both rhizobia and the nitrate [58,59,60] (see Figure 1). All these *CLE* genes in *L. japonicus* and *M. truncatula* systemically inhibit nodulation when overexpressed [54,56,58]. It was found that the NIN TF directly activates the expression of rhizobia-induced *CLE* genes in *L. japonicus* and *M. truncatula*, suggesting that NIN is responsible for the induction of AON [43,44]. Moreover, in *L. japonicus*, the *NIN* gene itself is under the negative regulation by AON [43]. In this relation, the “NIN-CLE-СLV1-like kinase” regulatory module resembles the “WUS-CLV3-CLV1” feedback regulation, which acts in the SAM, where the WUS (WUSCHEL) TF directly activates the expression of *CLV3* (encoding the CLE peptide) triggering signaling cascade to downregulate *WUS* expression by a feedback mechanism [164].

The nitrate-induced *CLE* genes in legumes were found to be activated by the NLP TFs [165,166], known as the master regulators of the nitrate-response in plants [31] (see also Section 2). Therefore, the activation of *CLEs* in response to rhizobia is mediated by NIN, whereas NLPs are responsible for the activation of *CLE* in response to the nitrate, mediating nitrate-mediated inhibition of the nodulation [43,44,166]. The presence of the NIN-specific binding sites together with NRE in the promoters of those genes which are activated by both NIN and NLP, brings together the rhizobia-induced and the nitrate-mediated pathways upregulating *CLE* expression and triggering the AON [166].

In addition to *CLEs*, NIN also activates the expression of the *MtCEP7* gene encoding a CEP (C-TERMINALLY ENCODED PEPTIDE) peptide [44] (see Figure 1). Like the CLE peptides, the CEP peptides represent a group of post-translationally modified regulatory peptides [167]. The *MtCEP1* and *MtCEP7* genes were found to be the positive regulators of nodulation [44,62]. In *A. thaliana*, *CEPs* are activated under nitrate deficiency, and they mediate the inhibition of the root growth under low nitrate condition [64]. For *CEPs* in *A. thaliana*, both local and systemic action has been described: the AtCEP1 peptide systemically, through a shoot-acting receptor, AtCEPR (CEP RECEPTOR), activates the expression of nitrate transporter genes in the root [65], whereas AtCEP5 inhibits LR development locally through a root-acting receptor [63]. In *M. truncatula*, MtCEP1 systemically stimulates nodule development through its receptor MtCRA2 (COMPACT ROOT SYSTEM ARCHITECTURE 2) in the shoot, and inhibits LR development, which was found to be mediated by a local MtCRA2 activity in the root [62,168,169]. However, recently, it was found that MtCRA2 might be involved in a negative regulation of nodulation in the root, since grafting experiments showed that plants with the *cra2* mutant roots had an increased nodule number at inhibitory nitrate levels [166]. Collectively, findings on CLE and CEP function in nodulation suggest that the systemic regulation of nodule number in legumes might have evolved based on the system that controls root architecture in plants in response to nitrogen availability.

Signaling cascades activated by the CEP and CLE peptides in the shoot were found to oppositely regulate a common downstream target, miRNA2111: CEP-activated signaling cascade activates *miRNA2111* in the shoot, whereas the CLE peptides through shoot acting receptor decreases the abundance of miRNA2111 in the shoot [170,171] (see Figure 1). miRNA2111 is a mobile regulator of the symbiosis moving from the shoot to the root, where it activates nodulation by targeting the transcripts of the *TOO MUCH LOVE* (*TML*) gene [170]. *TML* encodes an F-box protein which negatively regulates nodulation in the root [172], and it might be responsible for the ubiquitin-mediate degradation of a positive regulator of the symbiosis. However, the protein targets of TML have not been described up to date. Interestingly, in sweet potato *Ipomoea batatas*, miRNA2111 was found to be involved in wound response, and a wound-inducible gene, *IbFBK* (F-box/kelch repeat protein), was identified as its target [173]. Moreover, it was found that the kelch-repeat domain of IbFBK interacts with IbCNR8 (CELL NUMBER REGULATOR 8), which was suggested to lead to the ubiquitination and degradation of IbCNR8. CNRs are transmembrane proteins known as the regulators of plant organ size (see [174]). In soybean, the member of the CNR protein family, GmFWL1, has been identified, which exclusively is expressed in the root hair cells in response to rhizobia and in the nodules. RNAi knockdown of the *GmFWL1* gene resulted in a significant reduction in soybean nodule development, suggesting that it is a positive regulator of nodulation [175,176]. It is of great interest to learn if nodule-specific CNRs could interact with the F-box containing TML protein to be targeted for ubiquitin-dependent degradation, as it was suggested for FBK and its interacting protein of the CNR family in sweet potato.

It was also found that in rapeseed phloem sap miR2111 abundance was significantly increased under P starvation [91]. This suggests that long-acting miR2111 and its targets could play broader roles in plants, regulating root growth and architecture in response to nutrient availability. Moreover, the role of CLV1-like kinase and CLE peptides in phosphorus response was further shown in the arbuscular mycorrhizal symbiosis. In *M.truncatula*, the MtCLE53 and MtCLE33 peptides are induced by arbuscular mycorrhizal fungi and high phosphorus and negatively regulate root mycorrhization in *MtSUNN*-dependent manner, thereby mediating the autoregulation of mycorrhization [177,178]. Therefore, the CLV1-like kinase, encoded by the *MtSUNN* gene in *M. truncatula*, orchestrates different developmental responses in legumes by binding to different CLE peptide ligands.

## 7. Conclusions

The nitrogen-fixing symbiosis with rhizobia is a unique feature of legumes and *Parasponia* species from the *Cannabaceae* family. Regulatory pathways that are involved in the symbiotic nodule development have evolved based on the transcriptional regulators, many of which pre-exist in non-legume plants (see Figure 1). The close homologues of these regulators have also non-symbiotic function in plants and regulate different aspects of plant development, including LR development and root radial patterning, root and shoot meristem maintenance, flower transition, as well as the response to nutrient availability (see Table 1). Knowing which additional factors interact with such homologous regulatory modules in non-symbiotic condition will help to predict new players and new possible interactions in the regulation of the legume–rhizobia symbiosis.

Therefore, many of the regulators of nodule organogenesis also function in non-nodulating species, playing diverse roles in plant development. In this respect, what are the key features of legume-specific regulatory networks which control nodulation? NIN is a central regulator of the legume–rhizobia symbiosis, coordinating a set of conserved regulatory modules involved in nodule development. NIN itself has the homology with other NLP proteins. However, in the nodulating species, it lost nitrate-responsiveness and acquired *cis*-regulatory elements responsible for its expression in the root in response to rhizobia-induced and cytokinin-induced signaling pathways. Therefore, the NIN-coordinated regulatory network in nodulation might have evolved from the NLP-controlled network, which underlies nitrogen-dependent developmental processes in plants. In addition to this, the acquisition of the NIN-binding sites by a set of genes involved in rhizobial infection, LR development, and systemic response to nutrient availability made it possible to combine these regulatory modules in a single developmental program controlled by NIN, which has evolved to increase the specificity and effectiveness of symbiotic nodulation. Another set of evidence indicates that the nodulating species are characterized by a specific responsiveness of their root cortical cells to cytokinin. Recent findings suggest that a broader pattern of the *SCH-SCR* expression in the root cortex, due to the presence of the specific *cis*-regulatory elements in the *SCRs* promoters in legumes, could account for the ability of legume plants to activate cortical cell divisions in response to cytokinin treatment.

Therefore, during the evolution of legume plants, the changes in *cis*-regulatory elements in a set of genes, including *SCR* (broadening the expression pattern in the root cortex), *NIN* (acquisition of the rhizobia-induced and cytokinin-responsive elements (CE)), *LBD16,* and other targets of the NIN transcription factor (acquisition of the NIN-binding sites), might have been crucial evolutionary events necessary for the establishment of the nodule developmental program. Symbiotic nitrogen fixation is able to enrich agricultural ecosystems with a biologically available nitrogen and to minimize the application of nitrogen-containing fertilizers. The unraveling of evolutionary events underlying the establishment of the legume–rhizobia symbiosis is of great fundamental and practical interest, since it could help to introduce symbiotic features to non-legume species in the future.

## Figures and Tables

**Table 1:** The targets of the NIN transcription factor involved in the control nodule organogenesis.

Target Gene	Role in Nodulation	Other Functions Reported in Legume Plants	Examples of the Close Homologues in Non-Legume Species and Their Function
*LBD16/ASL18*	Stimulation of nodule primordium initiation [40,41]	Stimulation of LR primordium initiation [40,41]	LBD16/ASL18 stimulation of LR primordium initiation [45]
*NF-YA1* and *NF-YB1*	Regulation of rhizobial infection, stimulation of nodule primordia development, nodule meristem development, and tissue differentiation in the nodule [38,46]	Stimulation of LR development [37]	AtNF-YA2/AtNF-YA10 promotion of root growth and LR development [47]
*CRE1*	Activation of the cytokinin signaling cascade which stimulates nodule primordium formation [48]	Inhibition of LR development [48]	Activation of cytokinin signaling cascade which stimulates the shoot meristem activity, inhibits lateral root development [49,50,51], promotes root vasculature differentiation [49]
*miR172*	Downregulation of the NNC1 (AP2-like transcriptional repressor) which is involved in the inhibition of symbiotic gene expression due to the repression of NIN activity [42]	Root growth regulation in response to salt stress [52]	Downregulation of AP2/TOE, which act as negative regulators of flowering induction [53]
*MtCLE13/LjCLE-RS 1*,*2*	Systemic inhibition of nodule development (*MtCLE12,13,35/LjCLE-RS1*-*3/GmRIC*) [54,55,56];inhibition of nodule development in response to the nitrate (*MtCLE35/LjCLE-RS2,3/GmNIC*) [57,58,59,60]	-	AtCLE1/3/4/7 inhibition of LR development under nitrogen deficiency [61]
*MtCEP7*	Systemic stimulation of nodule development [44,62]	Inhibition of LR development [62]	Inhibition of LR development under nitrogen deficiency [63,64] systemic activation of the nitrate transporter genes in the root [65]
